# Incorporating weekly carboplatin in anthracycline and paclitaxel-containing neoadjuvant chemotherapy for triple-negative breast cancer: propensity-score matching analysis and TIL evaluation

**DOI:** 10.1038/s41416-022-02050-8

**Published:** 2022-11-17

**Authors:** Maria Vittoria Dieci, Luisa Carbognin, Federica Miglietta, Fabio Canino, Carlo Alberto Giorgi, Enrico Cumerlato, Ottavia Amato, Davide Massa, Gaia Griguolo, Elisa Genovesi, Giovanna Garufi, Diana Giannarelli, Antonio Tornincasa, Lucia Trudu, Silvia Michieletto, Tania Saibene, Marcello Lo Mele, Matteo Fassan, Giovanni Zarrilli, Federico Piacentini, Emilio Bria, Valentina Guarneri

**Affiliations:** 1 Department of Surgery, Oncology and Gastroenterology, University of Padova, Padova, Italy; 2 Division of Medical Oncology 2, Istituto Oncologico Veneto IRCCS, Padova, Italy; 3 Department of Woman and Child Health and Public Health, Fondazione Policlinico Universitario Agostino Gemelli, IRCCS, Roma, Italy; 4 Department of Medical and Surgical Sciences for Children and Adults, University Hospital of Modena, Modena, Italy; 5 Università Cattolica del Sacro Cuore, Roma, Italy; 6 Istituto Nazionale Tumori Regina Elena, Roma, Italy; 7 Breast Surgery Department, Istituto Oncologico Veneto IRCCS, Padova, Italy; 8 Surgical Pathology Unit, University Hospital of Padova, Padova, Italy; 9 Department of Medicine (DIMED), Surgical Pathology & Cytopathology Unit, University of Padua, Padova, Italy; 10 Istituto Oncologico Veneto IRCCS, Padova, Italy; 11 Comprehensive Cancer Center, Fondazione Policlinico Universitario Agostino Gemelli IRCCS, Roma, Italy

**Keywords:** Breast cancer, Imaging the immune system, Prognostic markers, Breast cancer, Tumour immunology

## Abstract

**Background:**

The generation of data capturing the risk-benefit ratio of incorporating carboplatin (Cb) to neoadjuvant chemotherapy (NACT) for triple-negative breast cancer (TNBC) in a clinical practice setting is urgently needed. Tumour-infiltrating lymphocytes (TILs) have an established role in TNBC receiving NACT, however, the role of TIL dynamics under NACT exposure in patients receiving the current standard of care is largely uncharted.

**Methods:**

Consecutive TNBC patients receiving anthracycline-taxane [A-T] +/− Cb NACT at three Institutions were enrolled. Stromal-TILs were evaluated on pre-NACT and residual disease (RD) specimens. In the clinical cohort, propensity-score-matching was used to control selection bias.

**Results:**

In total, 247 patients were included (A-T = 40.5%, A-TCb = 59.5%). After propensity-score-matching, pCR was significantly higher for A-TCb vs A-T (51.9% vs 34.2%, multivariate: OR = 2.40, *P* = 0.01). No differences in grade ≥3 haematological toxicities were observed. TILs increased from baseline to RD in the overall population and across A-T/A-TCb subgroups. TIL increase from baseline to RD was positively and independently associated with distant disease-free survival (multivariate: HR = 0.43, *P* = 0.05).

**Conclusions:**

We confirmed in a clinical practice setting of TNBC patients receiving A-T NACT that the incorporation of weekly Cb significantly improved pCR. In addition, A-T +/− Cb enhanced immune infiltration from baseline to RD. Finally, we reported a positive independent prognostic role of TIL increase after NACT exposure.

## Introduction

Triple-negative (TN) breast cancer (BC), as defined by the absence of hormone receptor expression and HER2 overexpression/gene amplification, accounts for 15% of all breast tumours and represents the most lethal BC subtype [1]. It is associated with a high risk of relapse, frequent visceral involvement, and short survival from the onset of metastatic disease [2, 3]. Neoadjuvant chemotherapy (NACT) followed by surgery is the standard approach for most early-TNBCs. The entity of tumour response after NACT provides important prognostic information for TNBC patients [4] as the achievement of a pathological complete response (pCR) after NACT is a strong surrogate of the long-term outcome at the patient level [5–7].

The inclusion of a platinum agent, especially carboplatin, in standard anthracycline-taxane-based NACT has been evaluated in randomised trials for TNBC [8–13]. A meta-analysis of published studies demonstrated significantly increased pCR with platinum-containing treatment as compared to platinum-free regimens from 37.0 to 52.1% [14]. However, the benefit of adding carboplatin to standard chemotherapy has been consistently suggested to come at the cost of a worse toxicity profile, especially in terms of haematological events. Therefore, based on the uncertainty regarding the impact of neoadjuvant carboplatin on long-term outcomes, the actual clinical value of this escalated neoadjuvant strategy has been the object of intense debate and international guidelines currently recommend the inclusion of carboplatin only after a careful balance of potential harms and benefits [15, 16]. However, recent findings from the Brightness trial, showing a significant improvement in EFS for Stage II/III TNBC patients resulting from the incorporation of carboplatin into a sequential paclitaxel-anthracycline neoadjuvant regimen (+/− veliparib) [17] will likely tip the scale in favour of considering the addition of carboplatin as a new standard of care in Stage II/III TNBC patients. These findings generated the urgency to further appraise the impact of implementing this escalated strategy in the clinic, where treatment schedules and doses are not always consistent with those applied in clinical trials. Moreover, there is the concern that the increased toxicity observed with the incorporation of platinum might be amplified in real-world populations, thus requiring dose adjustments, ultimately resulting in undermined dose density/intensity of anthracycline-taxane standard regimens.

Tumour-infiltrating lymphocytes (TILs) have an established role as a biomarker in TNBC patients undergoing NACT given their well-acknowledged association with relevant clinical outcome measures, gaining level I-b evidence for their clinical validity in this setting [18–20]. Indeed, baseline TILs have been consistently reported to be highly predictive of pCR achievement in TNBC undergoing NACT [18] and they have been reported to retain a positive impact on prognosis [21]. Additionally, TILs on RD have been associated with improved outcome in TNBC patients failing to achieve pCR after NACT [22, 23]. However, both these TIL-based biomarkers provide static information, thus possibly failing to capture the dynamics of the tumour immune milieu under NACT exposure [24, 25]. In fact, a mounting body of evidence supports the notion that chemotherapy may exert its antitumor activity, at least in part, via immune-mediated mechanisms, with several reports suggesting different chemotherapeutic agents, including anthracyclines, taxanes and platinum salts, being capable of priming anti-tumour adaptive immune responses [22, 26–28]. To date, several small breast cancer series reported an increase in TIL levels from baseline to residual disease (RD) in TNBC patients failing to achieve pCR after NACT [22, 29]. Nevertheless, there is still the need to better elucidate TIL dynamics under the pressure of NACT administration in TNBC cohorts homogenously receiving the current standard of care, namely anthracycline-taxane-based NACT with or without carboplatin.

In the present multicentric study, we evaluated the impact in terms of pCR rates, distant disease-free survival (D-DFS) and toxicity of the incorporation of weekly carboplatin into standard sequential anthracycline-taxane-based NACT for TNBC [30] by implementing a propensity-score matching to reduce the effect of confounding factors.

In addition, we described TILs changes from baseline biopsy to matched RD samples in TNBC patients not achieving pCR following anthracycline-taxane-based NACT with or without carboplatin, investigated the relative contribution of carboplatin in this regard and assessed the prognostic value of TILs dynamics.

## Methods

### Patient cohort

Consecutive patients diagnosed with TNBC and undergoing anthracycline-taxane-based NACT at three Italian Institutions (Istituto Oncologico Veneto-IRCCS, Padova; Policlinico Universitario Agostino Gemelli-IRCCS, Roma; AOUI—Modena) between 2006 and 2020 were identified. For the clinical cohort, only patients receiving sequential treatment with anthracycline-based chemotherapy and weekly paclitaxel (A-T) with/without weekly carboplatin (Cb) were included. TNBC status was defined as both hormone receptor-negative, (ER&PgR < 10%) and HER2-negative (IHC0/1+ or absence of *HER2* amplification by in-situ hybridisation). Clinicopathologic data, including age, stage, grade, primary BC ER, PgR and HER2 expression, proliferative index (ki67), treatment schedules, treatment-related toxicities, and clinical and pathological response to NACT were included in prospectively maintained Institutional databases.

### TIL assessment

Hematoxylin and eosin-stained (HES) slides from baseline biopsies and surgical specimens (in case of no-pCR) for each case were retrieved from the Institutional Pathology Archives. Stromal TILs levels were evaluated in a blinded fashion and in compliance with available recommendations for TILs assessment on untreated BC samples [31] and on residual disease after NACT [32]. Briefly, stromal TILs levels were defined as the percentage of the tumour stromal area occupied by mononuclear inflammatory cells—including lymphocytes and plasma cells—over the total intratumoral stromal area (polymorphonuclear leukocytes were excluded); TILs in areas with necrosis, crush artifacts, and inflammation around biopsy sites were excluded. TILs were considered both as a continuous and a categorical variable. In detail, TILs categories were defined as previously suggested [18]: low, 1–10%; intermediate, 11–59%, high, 60–100%. The changes of TILs from baseline biopsy to RD were reported as the mean delta and considered both as continuous and categorical variable. In particular, two categories were considered: TIL increase versus TIL no increase. Finally, we also computed the geometric means of the percentage of TILs to downsize the possible confounding impact deriving from large fluctuations and extreme values within small samples. The inter-rater agreement for TILs assessment among the three involved pathologists was evaluated on a sample of 78 cases using the Kappa statistic [33]. The agreement among readers was rated as follows: no agreement (*k* < 0), none to slight (*k* = 0.01–0.20), fair (*k* = 0.21–0.40), moderate (*k* = 0.41–0.60), substantial (*k* = 0.61–0.80), almost perfect *k* = 0.81–1.0027).

### Outcome definition

The main clinical objective of the study was to compare pCR rates between carboplatin-containing versus carboplatin-free neoadjuvant regimens.

The main translational objective was to assess TILs dynamics from baseline biopsy to RD following NACT exposure and to assess the prognostic value of this phenomenon.

pCR was defined as the absence of invasive disease from both breast and locoregional lymph nodes (ypT0/is ypN0). D-DFS was defined as the time from diagnosis to recurrence at a distant site or death from any cause. Patients without a D-DFS event were censored at the time of the last follow-up. To assess safety, haematologic adverse events were graded by adopting the Common Terminology Criteria for Adverse Events (CTCAE), according to the most recent version available at the time of diagnosis. Missed chemotherapy doses were also evaluated.

### Statistical analysis

Statistical analyses were carried out using SPSSv.27 and R3.6.122. Descriptive analyses were performed for the overall cohort and for the two cohorts (carboplatin-containing and carboplatin-free) separately. For continuous variables mean, median, quartiles and range values were computed. The distribution of continuous variables across groups were evaluated using Mann–Whitney *U* test, Kolmogorov–Smirnov, Wilcoxon and one-way analysis of variance (ANOVA) non-parametric tests. The distribution of categorical variables across groups was evaluated by applying the chi-squared test (*χ*^2^). In the clinical cohort, a propensity-score matching approach was applied to control selection bias. In particular, propensity-score matching represents a statistical method that, starting from a large reservoir of potential controls with many confounding variables, allows to produce a control group of modest size characterised by a similar distribution of covariates as compared to the treated group [34]. The selection of variables to be included in this model was driven by the weighting of true and potential confounders. True confounders were isolated among variables not equally distributed between carboplatin-treated and carboplatin-untreated cohorts; potential confounders were selected among those variables potentially affecting our primary outcome (pCR), or possibly affecting the treatment assignment in the clinical decision-making, or both. Among available variables, those selected for matching were: age, cT, cN, grade, histotype, BRCA status. Baseline tumour-infiltrating lymphocytes (TILs) were not used as matching variables given the high proportion of missing data, while Ki67 was not included given the lack of a centralised revision. A caliper width of 0.2 of the standard deviation of the logit of the propensity-score was applied for matching [35]. For consistency reasons, the same variables included in the propensity score were selected to be included in the logistic regression model. The binary logistic regression model was applied to calculate odds ratio (OR) for pCR between treatment groups.

The Kaplan–Meier method was used to estimate survival curves and the log-rank test was applied to test for differences between groups. The Cox regression model was used to calculate hazard ratios (HR) and 95% CI. All reported *P* values are two-sided, and the significance level was set at *P* < 0.05.

The study was approved by the ethics committee of participating centres, and all relevant ethical regulations have complied. Informed consent was obtained from all participants.

## Results

### Population

**Fig. 1:**
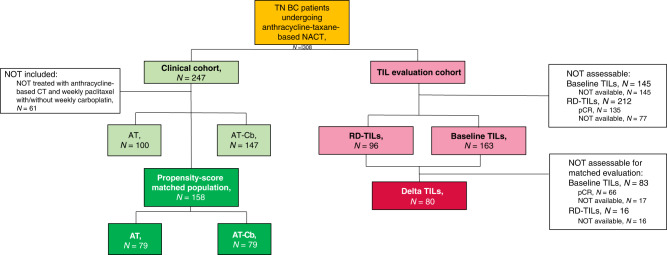
Flow diagram of the study. On the left: the clinical cohort of our study (*n* = 247) in which we identified the propensity-score matched population of patients treated with anthracycline-taxane (AT) chemotherapy with or without carboplatin (AT-Cb). On the right: the translational cohort were we evaluated tumor-infiltrating lymphocytes (TILs) at baseline (*n* = 163) and on residual disease (RD-TILs, *n* = 96), assessing the delta TIL variation.

The flow diagram of the study is shown in Fig. 1. Overall, 308 patients were included (clinical cohort and translational cohort). The clinicopathological features of the clinical cohort (*n* = 247) are shown in Supplementary Table 1.

### Propensity-score matched populations

Propensity-scores distribution and matching results are shown in Supplementary Fig. 1.

Clinicopathological characteristics of patients included in the propensity-score matched population (overall *n* = 158; AT, *n* = 79; AT-Cb, *n* = 79) are shown in Table 1.

**Table 1:** Clinicopathological features in the propensity-score matched population.

Clinicopathological features		TOT, *N* = 158		AT, *N* = 79		AT-Cb, *N* = 79		*P*
		*N*	%	*N*	%	*N*	%	
Age years, median (range)		54 (43–61)		51 (42–61)		54 (45–61)		0.495
Histotype	ductal/NOS	154	97.5%	77	97.5%	77	97.5%	1.000
	lobular/other	4	2.5%	2	2.5%	2	2.5%	
cT	T1	24	15.2%	11	13.9%	13	16.5%	0.904
	T2	112	70.9%	57	72.2%	55	69.6%	
	T3–T4	22	13.9%	11	13.9%	11	13.9%	
cN	Negative	80	50.6%	41	51.9%	39	49.4%	0.750
	Positive	78	49.4%	38	48.1%	40	50.6%	
Grade	G2	9	5.7%	6	7.6%	3	3.8%	0.303
	G3	149	94.3%	73	92.4%	76	96.2%	
Ki67 %, median (range)		60 (42–78)		60 (35–75)		65 (50–80)		0.172
TILs %, median (range)		13 (1–30)		10 (3–40)		15 (1–30)		0.899
*BRCA*	wt or unknown	134	84.8%	66	83.5%	68	86.1%	0.658
	Mut	24	15.2	13	16.5%	11	13.9%	

Clinicopathological features in the overall propensity-score matched population, and in the subgroups of  patients treated with anthracycline-taxane (AT, *n* = 79) and with the addition of Carboplatin (AT-Cb, *n* = 79).

Among clinicopathological variables not incorporated in our propensity-score matching model (TILs, and Ki67), no significant difference was observed between A-T and A-TCb cohorts.

**Fig. 2:**
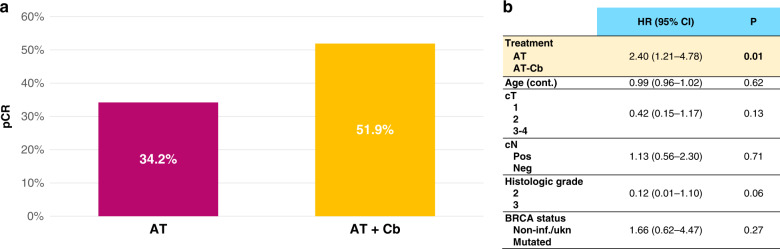
Pathological complete response (pCR) rates in propensity-score matched population. **a** Univariate analysis; **b** Multivariate analysis for pCR including treatment (A-T vs A-TCb), age (continuous), cT (cT1 vs cT2 vs cT3-4), cN (pos vs neg), histologic grade (2 vs 3), histotype (ductal/no-special type vs lobular/other special type), BRCA status (mutated vs non-informative or unknown). Data were analysed by multivariable logistic regression analysis.

As shown in Fig. 2a, after propensity-score matching, the rate of pCR was significantly higher for A-TCb vs AT. pCR rates were 34.2% vs 51.9% in the A-T vs A-TCb cohort (OR = 3.96, 95% CI 1.88–8.38, *P* < 0.001) in univariate analysis.

The significant impact on pCR of adding Cb to A-T was confirmed at multivariable logistic regression analysis corrected for matching variables (OR = 2.40, 95% CI 1.21–4.78, *P* = 0.01), as shown in Fig. 2b.

**Fig. 3:**
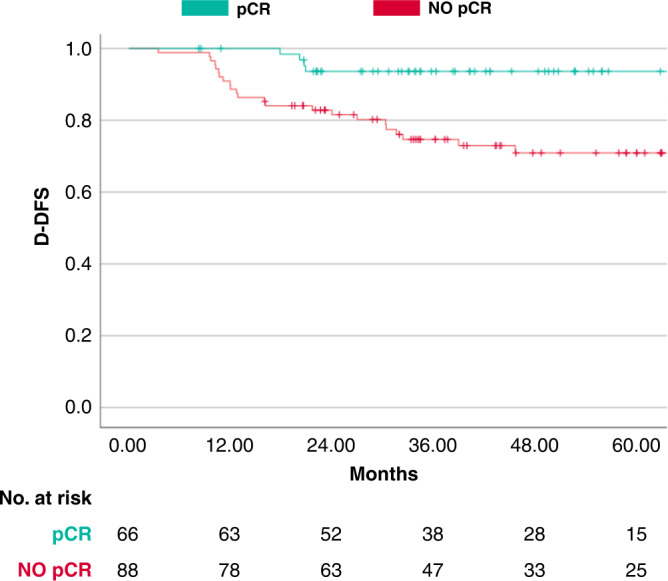
Kaplan–Meier curves for distant disease-free survival in the propensity-score matched population according to pathological complete response (pcR). Kaplan–Meier curves for distant disease-free survival (D-DFS) of achieving pCR versus NO-pCR.

Survival data were available for 156 patients. Overall, median duration of follow-up was 58.2 months (70.2 months in A-T and 45.4 months in A-TCb). When assessing the impact of adding Cb to A-T, no statistically significant difference in terms of D-DFS was observed, with 4-year D-DFS 80.2% vs 81.0% for A-TCb vs A-T, respectively (HR = 1.19, 95% CI 0.56–2.54, *P* = 0.647). By combining treatment groups, survival analysis according to pathologic response (pCR *n* = 68; RD *n* = 88) revealed a highly significant association between pCR and D-DFS, with 4-year D-DFS 93.6% vs 70.9% for pCR vs no-pCR, respectively (HR = 0.26, 95% CI 0.10–0.69, *P* = 0.007), as shown in Fig. 3.

### Toxicity

Rates of haematological toxicities are shown in Supplementary Table 2.

No significant differences in terms of Grade ≥3 haematological toxicities were observed between A-T vs A-TCb. A numerically higher rate of Grade ≥3 neutropenia was observed with A-TCb versus A-T (36.8% vs 28.0%), while A-T cohort was associated with numerically higher risk of febrile neutropenia as compared to A-TCb (5.6% vs 2.6%).

### Exploratory analysis

When comparing the choice of the anthracycline-taxane sequence (“*classical*” with anthracycline segment upfront and “*inverse*” with taxane segment upfront) between A-T versus A-TCb the strategy of inverting the sequence was most frequently adopted in patients receiving carboplatin as compared to patients not receiving carboplatin (82.3% vs 49.4%, *P* < 0.001). When focusing on the A-TCb cohort, patients treated with the inverse sequence received a numerically higher dose intensity of both taxane and carboplatin as compared to those treated with the classical sequence (median doses of taxane: 11 vs 9, *P* = 0.12; median doses of carboplatin: 10 vs 8, *P* = 0.10), with no impact on dose intensity of the anthracycline segment. When comparing pCR rates, a trend towards a higher likelihood of pCR was observed in A-TCb-treated patients receiving the inverse sequence as compared to the classical sequence (56.9% vs 28.6% *P* = 0.077). No difference in terms of haematological toxicity was observed according to the anthracycline-taxane sequence adopted.

In the propensity-score matched population, 19 (12%) patients exhibited ER-low phenotype. No significant difference in terms of pCR rates between ER-low and ER-zero cases was observed (*P* = 1.000). When separately assessing the impact of Cb incorporation in terms of pCR in ER-zero and ER-low, we observed higher pCR rates with A-TCb than AT in both cohorts (pCR rates AT vs A-TCb: ER-zero 34.8% vs 53.4%, *P* = 0.040; ER-low 30.8% vs 83.3%, *P* = 0.057).

### Tumour-infiltrating lymphocytes analyses

Baseline TILs and residual disease TILs (RD-TILs) were available for 163 patients and 96 patients, respectively. Clinicopathological features of the subgroups included and not included in the TILs analysis were not significantly different in terms of clinical stage, grade, histotype, carboplatin exposure and pCR rates (Supplementary Table IV).

The inter-rater agreement between readers was rated as almost perfect (*k* = 0.905, 95% CI 0.863–0.938, *P* < 0.001).

Mean and median values of baseline TILs were 16.6% and 5%, respectively; mean and median values of RD-TILs were 14.6% and 8%, respectively.

Levels of baseline TILs were not unbalanced between AT and A- TCb subgroup (*P* = 0.307).

#### Association of baseline TILs with pCR

Baseline TILs were significantly associated with pCR, in the overall population (pCR rates in low vs intermediate vs high TILs: 26.9% vs 54.3% vs 100%, respectively, *P* < 0.001) and in both A-T (pCR rates in low vs intermediate vs high TILs: 20.5% vs 35.0% vs 100%, respectively, *P* < 0.001) and A-TCb (pCR rates in low vs intermediate vs high TILs: 34.0% vs 69.2% vs 100%, respectively, *P* < 0.001) treatment subgroups, as shown in Supplementary Fig. 2. Considering TILs as a continuous variable, the significant association with pCR was confirmed in the overall population (*P* < 0.001) and in the A-TCb subgroup (*P* < 0.001), with a trend in the same direction in the A-T cohort (*P* = 0.088). These findings were confirmed when applying the geometric means of the percentage of baseline TILs.

#### TILs changes from baseline to RD

In the subgroup of patients failing to achieve pCR, matched evaluation of baseline and RD-TILs was available for 80 cases. In this cohort, mean and median values of baseline TILs were 9.3% and 5%, respectively; mean and median values of RD-TILs were 14.3% and 8%, respectively.

**Fig. 4:**
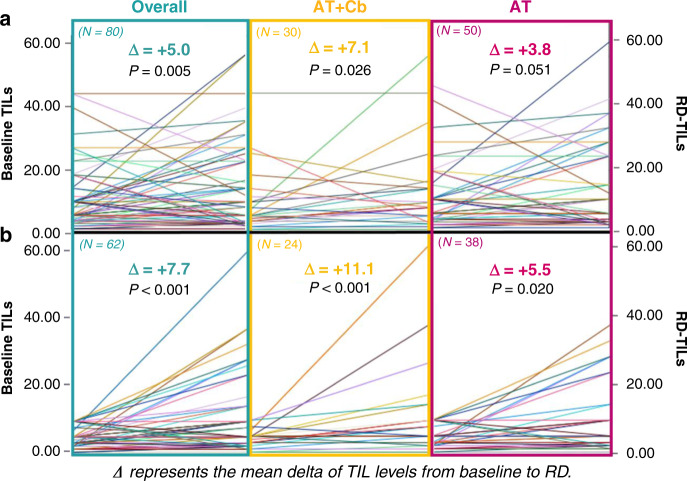
Matched baseline-residual disease TILs level in the overall and anthracycline + taxane (AT) + /− Carboplatin (AT-Cb) population. **a** Matched baseline-residual disease TIL level in the overall and anthracycline + taxane (AT) + /− Carboplatin (AT-Cb) population. **b** Matched baseline-residual disease TIL levels in the subgroup of patients with low baseline TILs. Data were analysed by Wilcoxon non-parametric test.

TIL levels significantly increased from baseline to matched RD in the overall population and in both A-TCb and AT treatment groups (with an association of borderline significance in the AT cohort), as shown in Fig. 4a. When considering patients with low baseline TILs, a significant increase of TIL levels was similarly observed in the overall population and in both AT and A-TCb subgroups (Fig. 4b). These findings were confirmed when applying the geometric means of the percentage of delta TILs.

The delta of TIL levels between baseline and RD was not significantly affected by carboplatin exposure in the overall cohort nor in the subgroup of patients with low TILs at baseline (*P* = 0.640 and *P* = 0.198, respectively). However, the magnitude of TIL increase after NACT appeared numerically greater after Cb exposure.

#### Prognostic effect of TILs: focus on TILs changes from baseline biopsy to RD

Baseline TILs were significantly associated with D-DFS (continuous variable, *P* = 0.010). However, when assessing the prognostic role separately in the pCR (*n* = 66) and no-PCR cohorts (*n* = 97), the significance of the association was lost (*P* = 0.11 and *P* = 0.70, respectively).

We did not observe a significant association between RD-TILs and outcome in terms of D-DFS (*P* = 0.171).

**Fig. 5:**
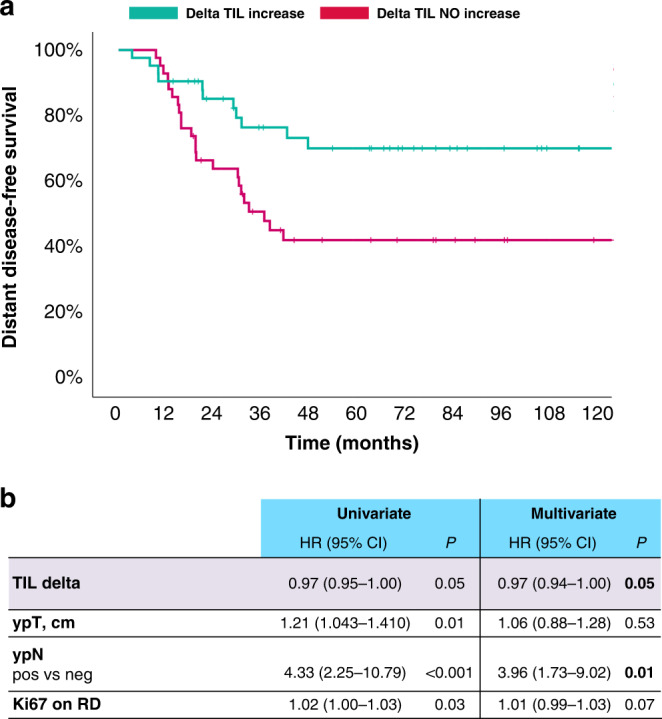
Kaplan-Meier curves and multivariate analysis for distant disease-free survival according to delta increase in tumor-infiltrating lymphocytes (TIL) from baseline biopsy to residual disease. **a** Kaplan–Meier curves for distant disease-free survival of TIL increase vs TIL NO increase. **b** Multivariate analysis for D-DFS including all variables significantly associated with D-DFS at the univariate analysis. Data were analysed by Cox regression model. TIL tumour-infiltrating lymphocytes, ypT post-neoadjuvant tumour size in centimetres, ypN post-neoadjuvant nodal status, RD residual disease, HR hazard ratio, CI confidence interval.

TILs increase from baseline to RD was positively associated with D-DFS both when considered a continuous variable (*P* = 0.05) and a categorical variable. In particular, 4-year D-DFS in the subgroups with TILs increase vs TILs no increase was 77.9% vs 41.7%, respectively; HR 0.35 (95% CI 0.16–0.76), *P* = 0.01, as shown in Fig. 5a.

At the multivariate analysis including all clinicopathological variables significantly associated with D-DFS at the univariate analysis, TIL delta and post-neoadjuvant nodal status were the only variables to maintain an independent significant prognostic value, as shown in Fig. 5b.

## Discussion

We investigated in a clinical practice scenario the impact in terms of antitumor activity, efficacy and safety of incorporating weekly carboplatin to standard anthracycline-weekly paclitaxel-based NACT in TNBC patients. After applying a propensity-score model to control selection bias, we observed an absolute 17.7% increase in pCR rates in the carboplatin-containing versus carboplatin-free cohort, corresponding to a relative OR of 2.40. This association was found to be independent at the multivariate analysis comprising other relevant clinicopathological variables, including BRCA status. This finding compares well with available evidence deriving from randomised trials which adopted various treatment schemes, especially regarding carboplatin schedule, with the 3-weekly administration representing the most widely adopted regimen. In addition, even when focusing on GeparSixto Phase II trial [10], which represents the only randomised study adopting a carboplatin-weekly schedule, other deviations like the incorporation of carboplatin into anthracycline+taxane-based concomitant chemotherapy, the adoption of non-pegylated doxorubicin as anthracycline component, and the omission of an alkylating agent, made this chemotherapy backbone mismatched to that routinely employed in clinical practice, thus precluding the possibility to perform specific comparisons with our study results. In the prospective single-arm Phase II trial by BMSO [13], Stage II/III TNBC patients received, similarly to our study, weekly carboplatin plus weekly paclitaxel, followed by epirubicin+cyclophosphamide, thus better reflecting a clinical practice setting. Interestingly, 54% of pCR was reported, thus making the carboplatin-driven pCR benefit observed in our study well integrated into this framework.

We did not observe significant differences in terms of D-DFS between carboplatin-containing versus carboplatin-free cohorts. However, the limited sample size and limitations related to retrospective design with lost-to-follow-up patients may have contributed to reducing the power of such survival analysis. So far, two randomised trials investigating the addition of carboplatin to anthracycline-taxane-based NACT captured a significant DFS/EFS benefit within the escalated arm, with a 44% and 37% relative reduction in the risk of DFS and EFS events in the GeparSixto [10] and Brightness trials [17], respectively. In addition, a recently updated meta-analysis of randomised trials evaluating the survival impact of incorporating carboplatin to NACT for TNBC revealed a statistically significant survival benefit in terms of EFS and a trend towards improvement of OS with carboplatin-based regimens over carboplatin-free NACT, thus possibly bringing to end the debate regarding the added value of including carboplatin to anthracycline-taxane-based NACT [36]. This consideration appears particularly well-timed also in the light of the KEYNOTE-522 Phase III trial results, which established the clinical value in terms of both pCR and EFS of adding neoadjuvant pembrolizumab to a chemotherapy backbone consisting of sequential carboplatin+paclitaxel and anthracycline, thus likely further promoting the implementation of carboplatin in this clinical setting [37].

As expected, when combining the two treatment cohorts, we confirmed the statistically significant and highly clinically meaningful association between pCR and D-DFS, thus further solidifying, in a clinical practice setting of TNBC patients receiving anthracycline-taxane-based NACT, the positive prognostic role of pCR at a single-patient level.

Despite what was expected based on available evidence, we did not observe a significant increase in the risk of developing haematological toxicity in the carboplatin-containing cohort as compared to the AT cohort. Indeed, safety data from the above-mentioned GeparSixto and BMSO trials, which best lend themselves to cross-study comparisons with our work in terms of carboplatin schedules and dosages, revealed unexpectedly high rates of haematological adverse events, with concerning rates of febrile neutropenia reported in the BMSO trial (28.6%). Under this scenario, findings from our multi-institutional study are reassuring and bring forts to the following consideration. Although the incorporation of carboplatin resulted in a slight reduction of the duration of the taxane segment, we observed fewer median doses of carboplatin than taxane, thus revealing the tendency, in a clinical practice setting, to spin off carboplatin from the taxane segment, despite the absence of meaningful toxicities, ultimately aiming at preserving taxane dose intensity, while, contextually, potentially resulting in the mitigation of the added harms of carboplatin. Of course, the impact of this phenomenon is not formally and accurately quantifiable, as well as hard to confirm or extrapolate from clinical trials where strict protocols for dose reductions/adjustments are usually embraced.

Exploratory analyses showed that the incorporation of carboplatin was significantly associated with a greater tendency of inverting the taxane-anthracycline sequence in the neoadjuvant setting. Interestingly in our work, within the A-TCb cohort, patients receiving the taxane segment-first were exposed to a higher taxane dose intensity, without negatively affecting the overall safety. In addition, those receiving the inverse taxane-anthracycline sequence experienced a trend towards increased pCR rates as compared to those receiving anthracycline upfront. These findings appear in line with available evidence suggesting that enhanced activity in terms of pCR may be obtained by administering the taxane segment prior to anthracyclines, with the improvement of taxane dose intensity—consistently observed with the adoption of this strategy—being put forward as a possible contributing factor. Of course, the interpretation of these results needs caution, and the clinical value of this observation deserves to be better elucidated in the context of properly powered studies.

In the translational segment of this study, we evaluated TILs dynamics from baseline biopsy to matched RD samples in TNBC patients not achieving pCR following anthracycline-taxane-based NACT and investigated the relative contribution of carboplatin in this regard. We observed a substantial increase of TILs levels after NACT exposure in the overall cohort and in both carboplatin-exposed and -unexposed subgroups. Previous reports similarly suggested a pro-immune effect of chemotherapy in TNBC patients treated in the neoadjuvant setting as surrogated by the observation of TILs level increase from baseline biopsy to RD [22, 28, 29, 38, 39]. However, it should be noted that available evidence mostly consists of small retrospective series of BC patients receiving various chemotherapy regimens. Importantly, we confirmed in a TNBC cohort homogeneously treated, that anthracycline-taxane-based NACT is capable of fostering tumour immune infiltration, and this effect was observed also in patients with immune cold tumour at baseline, thus solidifying the notion that chemotherapy can promote a more inflamed tumour-immune microenvironment by turning cold tumours into hotter ones. Notably, patients exposed to carboplatin experienced a similar NACT-induced immunomodulatory effect compared to those not receiving the platinum salt, with even a numerically greater TILs enhancement (this finding was not driven by an unbalance in terms of baseline TILs levels since they showed a similar distribution across AT and A- TCb subgroups). This finding well integrates within a previous observation suggesting that induction chemotherapy with platinum salts (or anthracyclines) is capable of determining an increase in T-cell infiltration and T-cell repertoire clonality as well as an upregulation of inflammation-related signatures [40]. Taken together, these observations, whilst overall consolidate the role of carboplatin as a valuable component of the chemotherapy backbone for TNBC patients undergoing neoadjuvant treatment, also outline A- TCb as the ideal partner for neoadjuvant immunotherapy.

Of note, we also observed a significant and independent positive prognostic role of TILs increase from baseline to RD in patients failing to achieve pCR, thus confirming similar findings from smaller cohorts of TNBC patients receiving diverse NACT regimens. This observation highlights that within the subgroup of TNBC patients defined at high risk of relapse based on the failure to achieve pCR, in those exhibiting TILs enhancement after NACT, the dismal prognostic effect of no-pCR may be mitigated. Conversely, patients with RD and failing to experience TIL increase after NACT may represent ideal candidates for escalated approach in the post-neoadjuvant setting. These observations hint that the evaluation of TIL dynamic changes after NACT exposure may result in a finer prognostic stratification of TNBC with RD.

Moreover, although baseline TILs were significantly and positively associated with prognosis in the overall cohort, this prognostic value was no longer significant when separately considering pCR and no-pCR subgroups. This observation might suggest that the positive impact on outcome of high TILs at baseline may reflect their ability to reliably surrogate for pCR achievement rather than an intrinsic prognostic value. Indeed, in our cohort we confirmed [18] a strong positive association between baseline TILs and pCR. Notably, all patients with high TILs at baseline subsequently obtained pCR, irrespectively from the receipt of carboplatin, thus outlining this biomarker as a potentially useful tool for the selection of patients who may be candidate for de-escalated approaches. In addition, in the subgroup of patients with RD, RD-TILs were not significantly associated with outcome. Although we acknowledge that the failure to confirm the already consolidated prognostic role of RD-TILs [22, 23] may reflect a statistical limitation, our findings, taken together, generate the hypothesis that the major immune-related determinant of prognosis in TNBC patients with RD, might actually be TIL dynamics under NACT exposure rather than the stationary caption of TIL levels on RD. Of course, our results deserve to be validated in larger and preferably prospective cohorts.

The present study has limitations. Its observational nature may be accountable for selection bias. However, a propensity-score matching approach has been adopted to control this limitation when assessing the association between carboplatin exposure and clinically relevant outcome measures (pCR and D-DFS). In addition, regarding the apparent neutral impact of the incorporation of carboplatin in terms of haematological toxicity, a possible role of carboplatin dose-capping, which is a commonly practised custom in clinics, especially in obese/overweight patients [41], cannot be excluded or neither quantified due to the absence of specific data regarding doses of each chemotherapy component.

Several strengths of the present study should also be acknowledged. Firstly, its multicentric nature contributed to increasing the robustness and generalisability of our results. Furthermore, we included TNBC patients homogeneously treated in terms of anthracycline-taxane backbone regimens. Finally, the TNBC population also included patients with 1-9% of ER expression. It has been consistently reported that tumours with the so-called ER-low phenotype share clinical features and behaviours more with “pure” TN [42, 43], thus fuelling a vivid debate regarding the most clinically relevant ER cut-off for defining TNBC. Importantly, both in our study and BMSO’s, TNBC was defined by adopting a 10% cut-off for ER negativity, thus generating two orders of considerations. Firstly, in clinical practice, the inclusion of carboplatin in the neoadjuvant management of BC appears to be often considered from ER 10% down, thus potentially mirroring the direction in which the ongoing academic debate on the optimal ER expression threshold for defining TNBC is leading in the clinical practice treatment-decision process [44, 45]. Secondly, the available evidence to which our study adds, underlines the urgency to generate solid data regarding the actual value of incorporating carboplatin to NACT in terms of pCR and survival in ER-low BC patients, given the current tendency in clinical practice to broaden the definition of TNBC also to include patients with ER expression 1–9%. Interestingly, the pCR enhancement resulting from the incorporation of Cb to AT appeared to be consistent across ER-zero and ER-low subgroups, with excellent pCR rates in ER-low patients receiving Cb (>80%). Although, as expected, ER-low cases represented only the minority of our entire cohort—thus imposing caution in the interpretation of results—this finding further highlights that ER-low BC retains a similar clinical behaviour to “pure” triple-negative BC also in terms of Cb sensitivity.

## Conclusions

In conclusion, we confirmed in a clinical practice setting of TNBC patients treated with anthracycline-taxane-based NACT that the incorporation of weekly carboplatin resulted in a substantial improvement of pCR rates, thus supporting the implementation of such escalated strategy in clinical practice. We also observed that AT +/− Cb was capable of enhancing immune infiltration from baseline biopsy to RD, and the such notable effect was also confirmed in patients with immune cold tumours at baseline. Finally, we reported a significant and independent positive prognostic role of TILs increase after NACT exposure, thus outlining this emerging biomarker as capable of refining the prognostic stratification of TNBC patients failing to achieve pCR after NACT.

## Supplementary information

Supplementary Material

EQUATOR network reporting guidelines

Reproducibility checklist

## Data Availability

The data that support the findings of this study are available upon reasonable request. The data are not publicly available due to privacy or ethical restrictions.
