# Correction: Incorporating weekly carboplatin in anthracycline and paclitaxel-containing neoadjuvant chemotherapy for triple-negative breast cancer: propensity-score matching analysis and TIL evaluation

**DOI:** 10.1038/s41416-022-02087-9

**Published:** 2022-12-02

**Authors:** Maria Vittoria Dieci, Luisa Carbognin, Federica Miglietta, Fabio Canino, Carlo Alberto Giorgi, Enrico Cumerlato, Ottavia Amato, Davide Massa, Gaia Griguolo, Elisa Genovesi, Giovanna Garufi, Diana Giannarelli, Antonio Tornincasa, Lucia Trudu, Silvia Michieletto, Tania Saibene, Marcello Lo Mele, Matteo Fassan, Giovanni Zarrilli, Federico Piacentini, Emilio Bria, Valentina Guarneri

**Affiliations:** 1 Department of Surgery, Oncology and Gastroenterology, University of Padova, Padova, Italy; 2 Division of Medical Oncology 2, Istituto Oncologico Veneto IRCCS, Padova, Italy; 3 Department of Woman and Child Health and Public Health, Fondazione Policlinico Universitario Agostino Gemelli, IRCCS, Roma, Italy; 4 Department of Medical and Surgical Sciences for Children and Adults, University Hospital of Modena, Modena, Italy; 5 Università Cattolica del Sacro Cuore, Roma, Italy; 6 Istituto Nazionale Tumori Regina Elena, Roma, Italy; 7 Breast Surgery Department, Istituto Oncologico Veneto IRCCS, Padova, Italy; 8 Surgical Pathology Unit, University Hospital of Padova, Padova, Italy; 9 Department of Medicine (DIMED), Surgical Pathology & Cytopathology Unit, University of Padua, Padova, Italy; 10 Istituto Oncologico Veneto IRCCS, Padova, Italy; 11 Comprehensive Cancer Center, Fondazione Policlinico Universitario Agostino Gemelli IRCCS, Roma, Italy

**Keywords:** Breast cancer, Imaging the immune system, Prognostic markers, Breast cancer, Tumour immunology

Correction to: *British Journal of Cancer* 10.1038/s41416-022-02050-8, published online 17 November 2022

The original version of this article contained an error in the funding section. This work was supported by RSF-2017-00000557 funding from Veneto Region (Italy). Role of the funder/sponsor: The funder had no role in the design and conduct of the study; collection, management, analysis, and interpretation of the data; preparation, review, or approval of the manuscript; and decision to submit the manuscript for publication. The original article has been corrected.

