# Supplementary material for: Incorporating weekly carboplatin in anthracycline and paclitaxel-containing neoadjuvant chemotherapy for triple-negative breast cancer: propensity-score matching analysis and TIL evaluation
Source: Br J Cancer. 2022 Nov 17;128(2):266–74. doi: 10.1038/s41416-022-02050-8 (PMC9902542; doi:10.1038/s41416-022-02050-8)
Supplement: Supplementary file 1 — Supplementary Material [file 41416_2022_2050_MOESM1_ESM.docx]

| **Clinicopathological features** | | **TOT, N=247** | | **A-T, N=100** | | **A-TCb, N=147** | | **P** |
| --- | --- | --- | --- | --- | --- | --- | --- | --- |
|  |  | **N** | **%** | **N** | **%** | **N** | **%** |  |
| **Age years, median (range)** | | 51 (43-60) | | 54 (43-63) | | 49 (42-58) | | 0.025 |
| **Histotype** | **ductal/NOS** | 234 | 94,7% | 96 | 96,0% | 138 | 93,9% | 0.605 |
|  | **lobular/other** | 13 | 5,3% | 4 | 4,0% | 9 | 6,1% |  |
| **cT** | **T1** | 44 | 17,8% | 14 | 14,0% | 30 | 20,4% | 0.192 |
|  | **T2** | 164 | 66,4% | 73 | 73,0% | 91 | 61,9% |  |
|  | **T3-T4** | 39 | 15,8% | 13 | 13,0% | 26 | 17,7% |  |
| **Stage** | **I** | 17 | 6,9% | 3 | 3,0% | 14 | 9,5% |  |
|  | **II** | 182 | 73,7% | 78 | 78,0% | 104 | 70,8% | 0.127 |
|  | **III** | 48 | 19,4% | 19 | 19,0% | 29 | 19,7% |  |
| **cN** | **negative** | 122 | 49,4% | 51 | 51,0% | 71 | 48,3% | 0.667 |
|  | **positive** | 125 | 50,6% | 49 | 49,0% | 76 | 51,7% |  |
| **Grade** | **G2** | 12 | 5,0% | 8 | 8,0% | 4 | 2,7% | 0.047 |
|  | **G3** | 224 | 90,6% | 85 | 85,0% | 139 | 94,6% |  |
|  | **NA** | 11 | 4,4% | 7 | 7,0% | 4 | 2,7% |  |
| **Ki67 %, median (range)** | | 60 (45-75) | | 60 (40-80) | | 65 (50-75) | | 0.328 |
| **TILs %, median (range)** | | 10 (5-35) | | 15 (5-40) | | 10 (5-30) | | 0,906 |
| ***BRCA*** | **wt or unknown** | 214 | 86,6% | 84 | 84,0% | 130 | 88,4% | 0.315 |
|  | **mut** | 33 | 13,4% | 16 | 16,0% | 17 | 11,6% |  |
| **Sequence CT** | **antra-tax (A-T)** | 87 | 35,2% | 53 | 53,0% | 34 | 23,1% | <0.001 |
|  | **tax-antra (T-A)** | 160 | 64,8% | 47 | 47,0% | 113 | 76,9% |  |
| **CT administration** | **Paclitaxel, median doses (Q1-Q3)** | 12 (9-12) | | 12 (10-12) | | 11 (9-12) | | 0.017 |
|  | **Antracycline, median cycles (Q1-Q3)** | 4 (3-4) | | 4 (4-4) | | 4 (3-4) | | 0.821 |

**Supplementary Table 1** - Clinicopathological features of the overall population

|  | **AT** | | **AT + Cb** | |
| --- | --- | --- | --- | --- |
| **Clinicopathological features** | **N (tot)** | **%** | **N** | **%** |
| **Leukopenia G3/G4** | 12 (75) | 16,0% | 13 (76) | 17,1% |
| **Neutropenia G3/G4** | 21 (75) | 28,0% | 28 (76) | 36,8% |
| **Febrile Neutropenia** | 4 (72) | 5,6% | 2 (76) | 2,6% |
| **Anemia G3/G4** | 1 (75) | 1,3% | 0 (74) | 0% |
| **Thrombocytopenia G3/G4** | 0 (75) | 0% | 0 (76) | 0% |

**Supplementary Table 2** - Rates of hematological toxicities in the propensity-score matched population

|  | Univariate | | Multivariate | |
| --- | --- | --- | --- | --- |
|  | HR (95% CI) | p | HR (95% CI) | p |
| TIL delta  Continuous  TIL increase vs NO TIL increase | 0.97 (0.95-1.00)  0.35 (0.16-0.76) | 0.05  0.01 | 0.97 (0.94-1.00)  0.43 (0.19-1.00) | **0.05**  **0.05** |
| ypT, cm | 1.21 (1.043-1.410) | 0.01 | 1.06 (0.88-1.28) | 0.53 |
| ypN  pos vs neg | 4.33 (2.25-10.79) | <0.001 | 3.96 (1.73-9.02) | **0.01** |
| Ki67 on RD | 1.02 (1.00-1.03) | 0.03 | 1.01 (0.99-1.03) | 0.07 |

**Supplementary Table 3** – Univariate and multivariate survival analysis for DDFS

**Supplementary Table 4** – Clinicopathological features of the subgroups included (TILs) and not included (NO TILs) in the TIL analysis.

| **Clinicopathological features** | | **TILs, N=163** | | | **NO TILs, N=145** | | **p** |
| --- | --- | --- | --- | --- | --- | --- | --- |
|  |  | **N** | **%** | | **N** | **%** |  |
| **Age years, median** | | 51 | | | 49 | | 0,846 |
| **Histotype** | **ductal/NOS** | 137 | 94,5 | | 153 | 93,9 | 1,000 |
|  | **lobular/other** | 8 | 5,5 | | 10 | 6,1 |  |
| **Stage** | **I** | 9 | 6,2 | | 8 | 4,9 | 0,271 |
|  | **II** | 108 | 74,5 | | 111 | 68,1 |  |
|  | **III** | 28 | 19,3 | | 44 | 27,0 |  |
| **Grade** | **G2** | 7 | 5,2 | | 11 | 6,8 | 0,632 |
|  | **G3** | 127 | 94,8 | | 150 | 93,2 |  |
| **Ki67 %, median** | | 60 | | | 65 | | 0,968 |
| **Carboplatin exposure** | **Yes** | 83 | | 57,2 | 78 | 47,9 | 0,110 |
|  | **No** | 62 | | 42,8 | 85 | 52,1 |  |
| **pCR** | **Yes** | 76 | | 52,4 | 97 | 59,5 | 0,250 |
|  | **No** | 69 | | 47,6 | 66 | 40,5 |  |


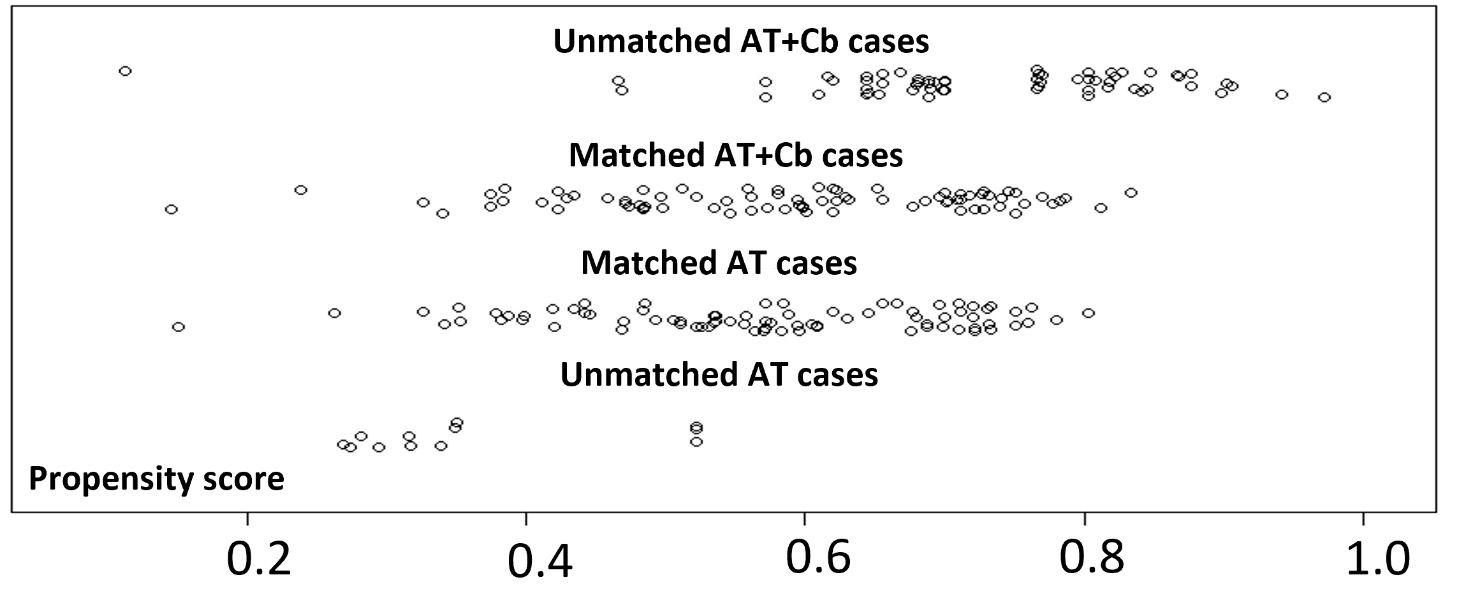


**Supplementary Figure 1** - Propensity-scores distribution and matching results


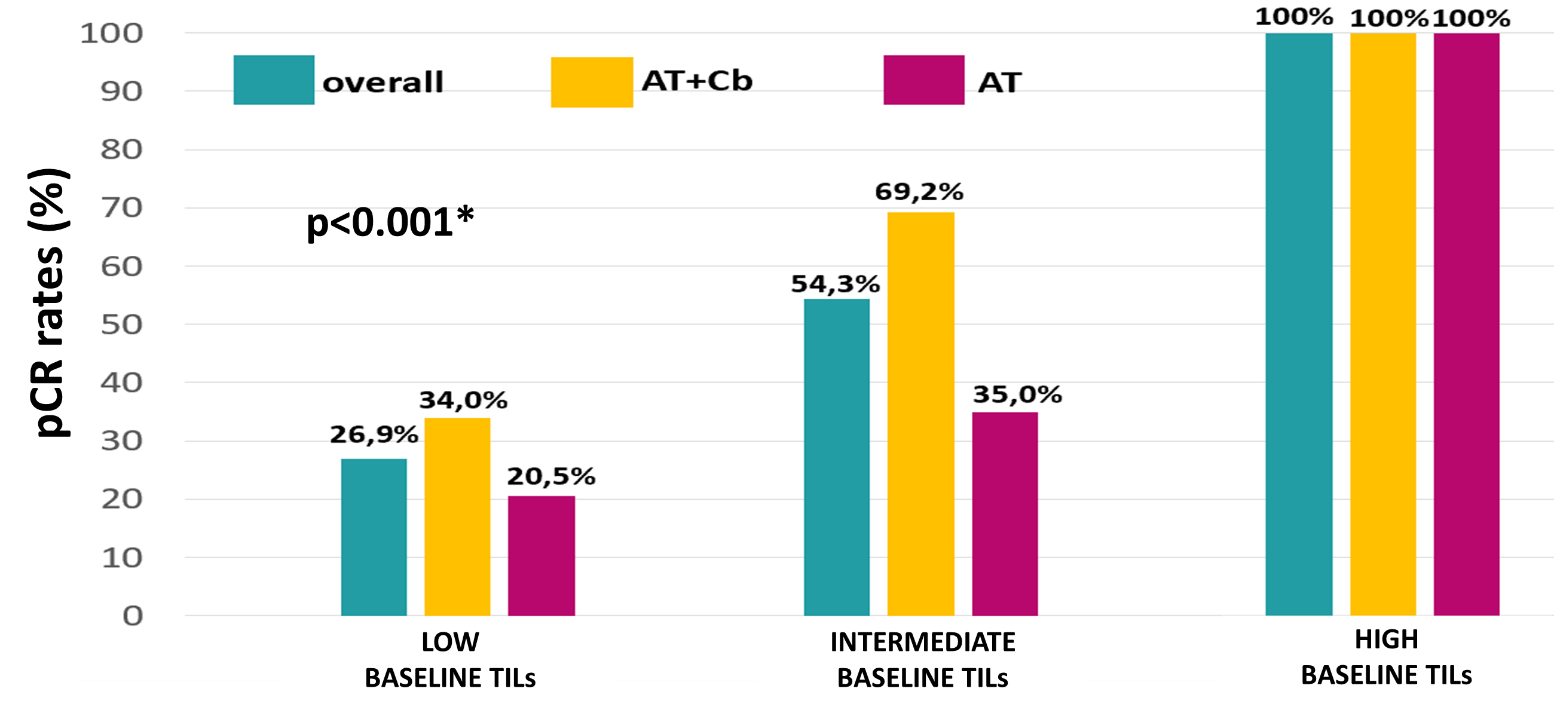


**Supplementary Figure 2** – pCR rates according to baseline TIL categories in the overall population and in treatment subgroups.

*significant association between pCR and baseline TILs when considered both as a continuous and categorical variable.
